# STAT3 precedes HIF1α transcriptional responses to oxygen and oxygen and glucose deprivation in human brain pericytes

**DOI:** 10.1371/journal.pone.0194146

**Published:** 2018-03-08

**Authors:** Robert Carlsson, Ilknur Özen, Marco Barbariga, Abderahim Gaceb, Michaela Roth, Gesine Paul

**Affiliations:** 1 Translational Neurology group, Department of Clinical Science, Wallenberg Neuroscience Center, Lund University, Lund, Sweden; 2 Department of Neurology, Scania University Hospital, Lund, Sweden; 3 Wallenberg Centre for Molecular Medicine, Lund University, Lund, Sweden; Texas Tech University, UNITED STATES

## Abstract

Brain pericytes are important to maintain vascular integrity of the neurovascular unit under both physiological and ischemic conditions. Ischemic stroke is known to induce an inflammatory and hypoxic response due to the lack of oxygen and glucose in the brain tissue. How this early response to ischemia is molecularly regulated in pericytes is largely unknown and may be of importance for future therapeutic targets. Here we evaluate the transcriptional responses in *in vitro* cultured human brain pericytes after oxygen and/or glucose deprivation. Hypoxia has been widely known to stabilise the transcription factor hypoxia inducible factor 1-alpha (HIF1α) and mediate the induction of hypoxic transcriptional programs after ischemia. However, we find that the transcription factors Jun Proto-Oncogene (c-JUN), Nuclear Factor Of Kappa Light Polypeptide Gene Enhancer In B-Cells (NFκB) and signal transducer and activator of transcription 3 (STAT3) bind genes regulated after 2hours (hs) of omitted glucose and oxygen before HIF1α. Potent HIF1α responses require 6hs of hypoxia to substantiate transcriptional regulation comparable to either c-JUN or STAT3. Phosphorylated STAT3 protein is at its highest after 5 min of oxygen and glucose (OGD) deprivation, whereas maximum HIF1α stabilisation requires 120 min. We show that STAT3 regulates angiogenic and metabolic pathways before HIF1α, suggesting that HIF1α is not the initiating trans-acting factor in the response of pericytes to ischemia.

## Introduction

In the brain, pericytes surround the entire microvasculature and take an important role in the neurovascular unit [1,2] by maintaining homeostasis in both, the developing and adult brain [3,4]. Pericytes respond quickly to external signals such as hypoxia, and recent data show that they secrete pro- and anti-inflammatory factors, and angiogenic molecules when exposed to oxygen or/ and glucose deprivation [5–8]. We have previously demonstrated hypoxia as a potential trigger to mobilize and recruit pericytes into tumors, where they associate with the tumor vasculature [9]. Similarly, ischemia (constituting oxygen and glucose deprivation) activates pericytes, but also regulates their differentiation and secretory properties [10] [11]. Little is known, however, about the transcriptional regulation of the response of brain pericytes to different hypoxic conditions, which might provide information on potential therapeutic targets to improve stroke outcome or reduce tumour vascularisation. Interestingly, in stroke mouse models, precondition treatment with hypoxia has been shown to reduce the infarct volume and detrimental outcome of subsequent stroke [12].

In the literature, hypoxia-inducible transcription factor (HIF-1) has long been considered the most important transcriptional regulator of cellular responses to hypoxia [13]. While the induction of the responses to hypoxic/ischemic stress induces the stabilisation of the transcription factor HIF1α, little it is known about the effect of hypoxia on Signal Transducer and Activator of Transcription-3 (STAT3) signalling [14,15]. STAT3 is a transcription factor activated in response to interleukin 6 (IL6) that regulates its transcriptional activity by inducing phosphorylation, homo-dimerization and nuclear translocation of STAT3 homodimers [16]. Interleukin 6 is important for protection against detrimental effects and for recovery in a stroke mouse-model [17,18] and activated STAT3 regulates Vascular endothelial growth factor expression (VEGF), a growth factor important for angiogenesis after stroke [19]. Activated STAT3 has been described as a positive regulator of HIF1α [20,21]. Indeed, STAT3 phosphorylation increases expression of HIF1α by inhibiting HIF1α degradation in tumor cells [22]. However, it remains unknown how STAT3 signalling and its interaction with HIF1α is modulated in pericytes exposed to hypoxic and ischemic conditions.

In this study, we present a bioinformatic analysis of the transcriptome of human brain pericytes after exposure to a) glucose deprivation, b) hypoxia (0.5–0.7% oxygen) and c) combined oxygen and glucose deprivation (OGD). Our analysis provides evidence of different gene expression profiles of human brain pericytes subjected to hypoxia with or without glucose in the favour of genes involved in cell metabolism, apoptosis and angiogenesis. Using a set of ChIP-seq analyses, we show that STAT3 occupied target genes are regulated before the HIF1α target genes. Lack of glucose in the absence of oxygen emphasises STAT3 activity, but does not have the same relative impact on HIF1α. We show that hypoxic or OGD treated pericytes respond to the treatments by transcriptional programs initiated primarily by STAT3, c-JUN and NF-κB, and not HIF1α and we confirm this temporal regulation at protein level. We further analyse STAT3 regulated genes and show that they involve angiogenic and metabolic pathways. These findings have implications for modulating pericyte activity early in ischemic stroke, and identify a set of regulated genes that are targets of STAT3, prior to the induction of HIF1 regulated genes.

## Material and methods

### Human brain pericytes

We previously established and characterized an adult human brain pericytes line, obtained from brain tissue harvested from individuals undergoing ventriculostomy or surgery for intractable temporal lobe epilepsy as described [23]. All procedures were performed with informed written consent by the patient for the donation of brain tissue and approved by the ethical committee of the Scania University Hospital, Lund, Sweden. Using flow cytometry, our previous studies show that human brain pericytes express the key pericyte markers including PDGFRb (CD140b), CD146, CD105, and CD13[24] [25]and are negative for monocyte/macrophage markers CD14, the microglial marker CD11b and the endothelial marker CD31[10,24,25]. The human brain pericytes were expanded in Stemline medium (Sigma-Aldrich) supplemented with 2% fetal bovine serum (FBS, Invitrogen), 1% Penicillin/Streptomycin (P/S, Gibco), 20 ng/ml basic fibroblast growth factor (bFGF, Invitrogen) on gelatin coated culture flasks (Nunc) and incubated at 37°C in 5% CO_2_ conditions (Heraeus HERAcell 150 CO_2_ incubator, Thermo Scientific). The cells grew exponentially with a doubling time of approximately 48 hs, reaching approximately 85% confluence after 48-72hs. Commercially available pericytes (ScienCell) were expanded in pericyte medium (ScienCell) according to the manufacturers instructions.

### Oxygen and/or glucose deprivation

The cultures were incubated in defined medium for 24 hs at 37°C in 5% CO_2_ and then washed with phosphate buffered saline (PBS). First, cells were exposed to oxygen deprivation (0.5–0.7% oxygen) conditions generated in a humidified, gas-tight hypoxia chamber incubator (Electrotek) with a gas composition of 85% N_2_, 5% CO_2_ and 10% H_2_ [26]. The OGD or hypoxic media were pre-bubbled in the hypoxic chamber for 30min resulting in 0.5–0.7% O_2_ when added to the cells. During experiments, an indicator solution was placed in the chamber. Oxygen tension was measured by an oxygen probe and oxygen levels were below 1 mmHg.

For total RNA isolation and immunocytochemistry cells were cultured in serum-free Dulbecco's Modified Eagle Medium (DMEM, Invitrogen) either with or without glucose at 37°C, over several time points: 5min, 2hour (h), 6h. Control samples were incubated under normoxic conditions with oxygen and glucose (5% CO_2_ at 37°C).

### Microarray data analysis

Samples were hybridized to Human HT-12 v4.0 Expression BeadChips (Illumina Inc.) at the SCIBLU Genomics Center at Lund University, Sweden (www.lu.se/sciblu). Microarray data were initially pre-processed and normalized using the quantile normalization method [27]. These analyses were performed using GenomeStudio software V2011.1. Illumina data was deposited in the NCBI GEO database with the accession number (GSE109233). Non-annotated probe sets and probe sets with signal intensities below the median of the negative control intensities in 80% of the samples that were not belonging to one condition were excluded. The remaining probe sets were used to construct box plots (Prism, version 6), PCA-plots of the top 500 probe sets p<0.05 to non-treated control (R, [28] and Venn-diagrams fold change (fc) >1.5, p<0.05 (R, [29]).

### Gene set enrichment analysis

Gene set enrichment analysis (GSEA) was performed on 14200 probe sets obtained from the initial cut-off analysis of the Illumina data where genes that were unexpressed in all the conditions were excluded [30,31]. The 14200 probe sets were analysed according to default settings in the GSEA software to identify Hallmark pathways regulated in glucose deprivation, hypoxia or OGD compared to the control condition.

### Identification and gene ontology (GO) analysis of bound regulated genes

In order to identify bound regulated genes in the Illumina gene expression data set, ChIP-seq data for c-JUN (wgEncodeEH000719), NF-κB (wgEncodeEH000690), STAT3 (wgEncodeEH001799) from the Encode consortium [32,33] and HIF1α [34] (JNSH) were downloaded. For the HIF1α ChIP-seq data a MACS2 analysis was made with a p-value <0.05 to identify binding sites and a. BED file was extracted in Galaxy [35]. The genes corresponding to the genomic coordinates within 5Kbp of a transcriptional start site (TSS) in the JNSH ChIP-seq data were then identified with Galaxy-Cistrome [36]. The Illumina probe-set expression list was collapsed to contain individual genes keeping only the highest value of multiple probe sets in R. The 5Kbp TSS genes were filtered against a gene list of Illumina gene expression data to generate a list with expression values from pericytes with peaks within 5Kbp of the TSS for each of the JNSH transcription factors. Each of the lists for the JNSH transcription factors was then filtered for expression of regulated genes at an fc>1.5 in the hypoxia or OGD conditions, respectively. These data were then used to identify bound regulated genes (BRGs) from the Illumina data. Binding site data from the ChIP-seq data were compared to the expression of genes of a fold change (fc) >1.5 of the Illumina data set and BRGs were extracted for each transcription factor. The JNSH fc>1.5 lists were used to construct Venn diagrams in R with the VennDiagram-package [29]. The BRGs of STAT3 and HIF1α were imported into the Python software and analysed with the default settings except that all GO:terms used in the analysis were from: GO database version 1.2, released 2017-12-27 [37].

### QPCR

Independent triplicates (n = 3) of 44.000 human brain pericytes were cultured in 4 well plates (24-plate well size) and grown for 20h. The cells were then washed with PBS. Cells were incubated in normoxia, hypoxia or OGD as described above for 2 or 6 hs, respectively. Then 300μl of RLT-plus-lysis buffer (Qiagen) was added. The lysate was frozen in -20°C prior to RNA isolation. Total RNA was isolated using the Qiagen RNA mini plus kit and protocol. A Nanodrop 3000 was used to measure the quality and quantity of the RNA. The RNA was then reverse transcribed with the iScript RT-PCR kit (BioRad) with reactions containing reverse transcriptase (RT) and without RT (-RT). The cDNA was diluted from 20μl to 150μl to be able to load 5μl cDNA per qPCR reaction. QPCR was run in 20μl reaction on a BioRad qPCR-machine with the SSO-advanced qPCR-SYBR mix according to the manufacturers instructions (BioRad). The fold changes between treatments were normalised to the B2M house keeping gene shown to be stable in hypoxic conditions [38,39]. The primers for the qPCR were designed by primer 3 in the NCBI primer selection software and are available on request. Primers were from TAGC Copenhagen. The qPCR data was analysed in Graphpad Prism and statistic significance was assessed with 2-way ANOVA and Dunnett’s multiple comparison test.

### Western blot

For western blot, human brain vascular pericytes (ScienCell) were seeded at a density of 170.000 cells per 6 well plate and grown for 20 hs in a cell culture incubator and washed in PBS before normoxic, hypoxic or OGD treatment, respectively. The cells were incubated in OGD media for OGD, and OGD media plus glucose for the normoxic and hypoxic conditions [26]. After 15, 45, 75 or 120 min, 350μl lysis buffer (0.125 M Tris-HCl pH 6.8, 1% SDS, with protease- (Thermo Scientific) and phosphatase-inhibitors (Thermo Scientific)) was added directly to the cells (whole cell lysate). The lysates were sonicated for 10s at an amplitude of 10% in a sonicator (Qsonica) to disrupt genomic DNA. For nuclear extracts, pericytes were hypotonically swelled and the plasma membrane disrupted with NP-40 to liberate the cytoplasm from the nuclei according to [40]. The Nuclei were then lysed in 50μl of a high salt buffer containing PMSF [40]. 1xSDS-load buffer was added to an aliquot of the nuclear extracts and the whole cell lysates, and run on BioRad 15 well 4–15% SDS-PAGE gels (BioRad) for western blotting onto Turbo-transfer-packs (BioRad). The post-transferred nitrocellulose membranes were then blocked in 5% milk in 1xPBS with 0.1% Tween-20 (PBS-T). The membranes where then incubated with primary antibodies rabbit anti-pS727-STAT3 (1:10000, Abcam) for 2h or mouse anti-STAT3 (1:1000, Cell Signalling technology (CST)) in 5% BSA tris-buffered saline (TBS) with 0.1% Tween-20 (5%BSA-TBS-T). Anti-pY705-STAT3 (1:1000, CST) or rabbit anti-HIF1α (1:1000, CST) was incubated in 5%BSA-TBS-T. The membranes were washed x3 in PBS-T for 5min and rabbit-anti-mouse-HRP antibodies (1:10000, Dako) or goat-anti-rabbit-HRP antibodies (1:5000, Dako) were added and incubated for 1h at room temperature on a rotator. The membranes were washed x3 5min in PBS-T and specific immunoreactivity was visualised by adding the HRP-substrates Clarity or Clarity max (BioRad) and measuring the chemiluminescence on a Chemidoc (Biorad).

### Immunocytochemistry

Briefly, 10.000 cells were seeded on coated coverslips with human fibronectin (1μg/ml) and vitronectin (0,25 μg/ml) in 24 well plate wells and incubated at 37°C for 20 hs. The media was exchanged for PBS just before the hypoxic experiment. Normoxia, hypoxia and OGD were performed as described above. All treatments were performed for 5 or 120min.

Cells were stained in the 24 well plates with coverslips. Cells were fixed in the media with 8% paraformaldehyde (PFA) in PBS. Unspecific binding was blocked by the addition of block buffer (1xPBS, 5% normal rabbit serum, 0.3% Triton X-100) for 60 min. The pS727-STAT3 (Abcam) and the HIF1α-antibody (CST) were diluted 1:800 in antibody dilution buffer (ADB) (1X PBS, 1% BSA, 0.3% Triton X-100) and incubated with the cells for 16h at +4°C. The secondary antibody anti-rabbit-Cy3 (1:500, Jackson) was diluted in ADB and incubated for 1 h with the cells after which Phalloidin-647 (Abcam) in 1% BSA, 1xPBS was added for 1h at room temperature in the dark.

Confocal pictures were acquired with a Leica TCS SP8 confocal microscope. 15 z-stacks with 1μm intervals were taken in 3 random areas with a 20x objective. Pictures were analysed in with ImageJ by manually outlining the cell nucleus and measure the intensity of the signal. Cells with obvious apoptotic shape were excluded from the quantification. Negative control staining was used to calibrate the intensity values. The average of all measured cells was normalized to the respective 5min normoxic condition.

## Results

### Gene expression analysis: the hypoxic response is transcriptionally activating rather than silencing

**Fig 1:**
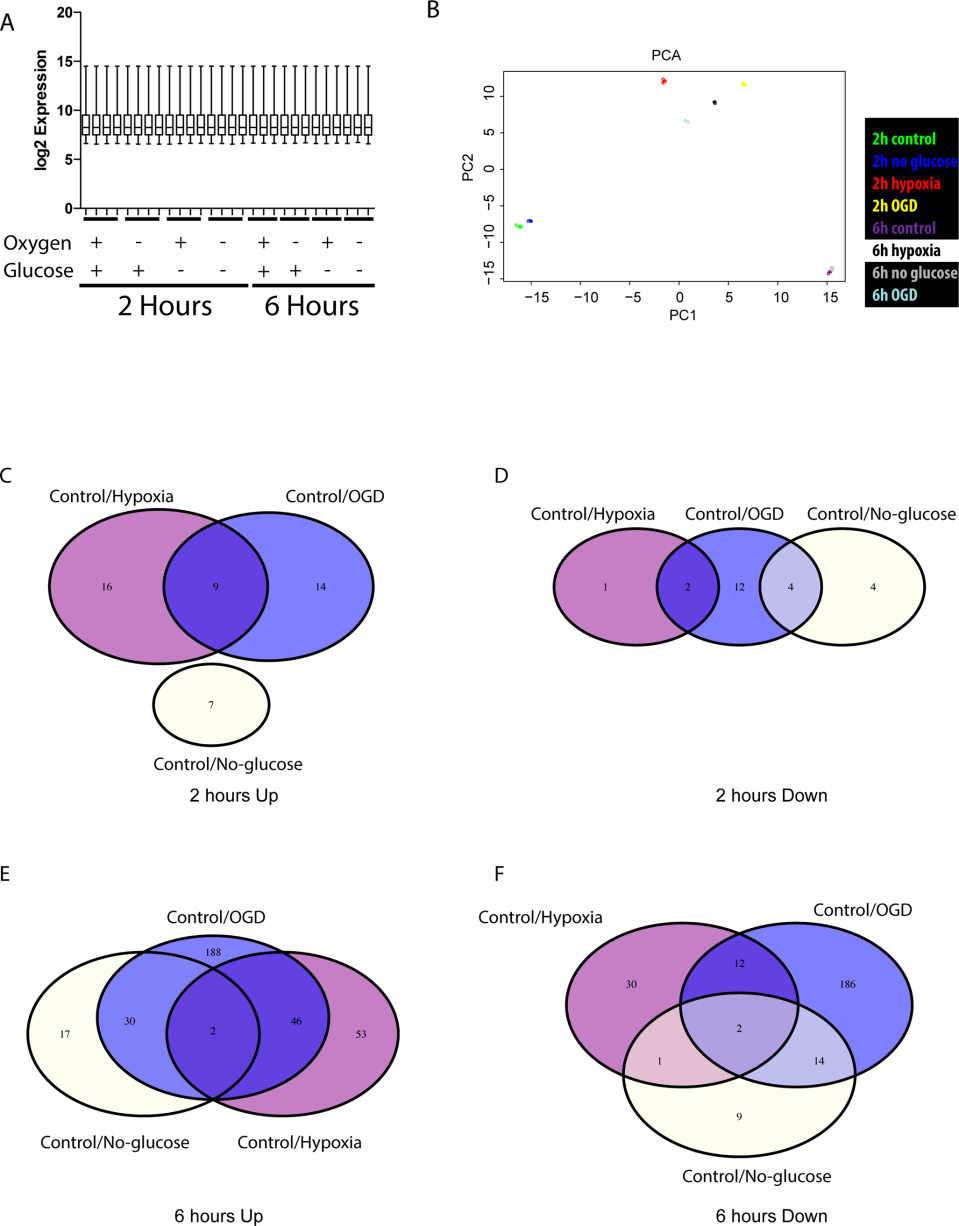
OGD produces a stronger gene regulatory response than hypoxia and glucose deprivation alone. (A) Normalised log2 transformed gene expression data was compared in a box plot to ensure that normalisation was equal between the samples of the bead-array. Error bars show standard deviation (SD). (B) PCA-plot of the top 500 probe sets p<0.05 comparisons to control to show that the replicates within the gene expression data group according to treatments. (C, E) Venn diagrams were constructed from the upregulated genes, (C) fold change (fc) >1.5 with a p-value <0.05 after 2hs or (E) 6 hs of: Control, Hypoxia, or oxygen and glucose deprivation (OGD). (D, F) Venn diagrams of down regulated genes fc<0.66, p-value <0.05 in (D) 2h or (F) 6 h treatments as in (C, E).

Using an Illumina platform, we first performed gene expression profiling containing more than 47,000 probes on human brain pericytes after exposure to glucose deprivation, hypoxia or OGD for 2 or 6h, respectively. A box plot showed that the normalisation of the individual probe sets was very similar (Fig 1A), whereas a principal component analysis grouped the replicates according to their treatments, respectively (Fig 1B). The resulting lists for up- or down-regulated genes for the 2 or 6h treatments were then plotted in Venn-diagrams (Fig 1C–1F). When comparing the data from 2h treatments it became evident that the hypoxic or glucose deprivation conditions affects similar responses in terms of how many genes are affected in the different treatments, except the hypoxic condition with glucose that has 25 probe sets upregulated at 2h, but only 3 probe sets are downregulated in the same condition. This indicated that the hypoxic response was a transcriptionally activating rather than transcriptionally silencing response (Fig 1C: Hypoxia-Up compared to Fig 1D: Hypoxia-Down). The same trend was seen in the data from the 6h treatments, hypoxia initiates upregulation of 101 probe sets, whereas only 45 probe sets were downregulated, respectively. This led us to examine whether specific transcriptional activators were predominantly active in the hypoxic condition. When comparing the 2h to 6h treatments it was clear that the 6h treatment evokes the regulation of more probe sets than the 2h treatment (Fig 1C and 1D to Fig 1E and 1F). Moreover, the effect of OGD treatment (having in addition to hypoxia also glucose deprivation) was generally inducing a stronger response at 2h, except upregulated probe sets at 2h of hypoxia (14 OGD/ 16 hypoxia), but also a much stronger response in terms of downregulated genes (12 OGD/ 1 hypoxia). The stronger effect of the OGD treatment was further emphasised at 6h of treatment. There, OGD upregulated 188 probe sets whereas the hypoxic treatment upregulated only 53 probe sets. The 186 downregulated probe sets in the OGD condition compared to 30 probe sets in the hypoxic condition, again suggesting that the hypoxic condition was initiating a response that was upregulating transcription rather than suppressing it.

### Gene set enrichment analysis of gene expression

Small changes in mRNA expression of many genes collectively contribute significantly to cellular decisions, and provide cues as to how these genes are regulated by transcription factors. To study the effects of any, small or big, changes in gene expression, a gene set enrichment analysis (GSEA) was performed. In the GSEA analysis it was evident that pericytes treated with hypoxia responded with upregulation of genes normally upregulated in hypoxia, and that the absence of glucose triggers the genes of the epithelial to mesenchymal transition (EMT) (Tables 1–6). The top hits of the GSEA analysis with NFκB signalling through tumour necrosis factor (TNF) as the major finding in both the hypoxic condition and OGD conditions at 2 and 6h suggested that HIF1α may not be the only transcription factor active in the hypoxic and OGD conditions.

**Table 1:** Upregulated gene sets after 2h hypoxia.

NAME	NOM p-val	FDR q-val	FWER p-val
HALLMARK TNFA SIGNALING VIA NFKB	0.00	0.00	0.00
HALLMARK HYPOXIA	0.00	0.00	0.00
HALLMARK INFLAMMATORY RESPONSE	0.00	0.00	0.00
HALLMARK KRAS SIGNALING UP	0.00	0.00	0.00
HALLMARK COMPLEMENT	0.00	0.00	0.00
HALLMARK IL6 JAK STAT3 SIGNALING	0.00	0.01	0.03
HALLMARK INTERFERON GAMMA RESPONSE	0.00	0.01	0.03
HALLMARK ALLOGRAFT REJECTION	0.00	0.02	0.13
HALLMARK APOPTOSIS	0.00	0.02	0.14
HALLMARK IL2 STAT5 SIGNALING	0.00	0.02	0.17
HALLMARK EPITHELIAL MESENCHYMAL TRANSITION	0.00	0.03	0.20
HALLMARK COAGULATION	0.01	0.03	0.21
HALLMARK INTERFERON ALPHA RESPONSE	0.01	0.03	0.25
HALLMARK UV RESPONSE UP	0.01	0.06	0.45
HALLMARK G2M CHECKPOINT	0.02	0.08	0.58
HALLMARK GLYCOLYSIS	0.02	0.08	0.63
HALLMARK TGF BETA SIGNALING	0.07	0.09	0.68
HALLMARK P53 PATHWAY	0.02	0.09	0.71
HALLMARK ESTROGEN RESPONSE LATE	0.04	0.10	0.77
HALLMARK ANGIOGENESIS	0.12	0.12	0.85

**Table 2:** Upregulated gene sets after 2h no glucose.

NAME	NOM p-val	FDR q-val	FWER p-val
HALLMARK COAGULATION	0.01	0.13	0.15
HALLMARK EPITHELIAL MESENCHYMAL TRANSITION	0.00	0.14	0.29
HALLMARK APICAL JUNCTION	0.02	0.18	0.50
HALLMARK ADIPOGENESIS	0.00	0.15	0.55
HALLMARK BILE ACID METABOLISM	0.08	0.23	0.78
HALLMARK ANDROGEN RESPONSE	0.06	0.20	0.79
HALLMARK HEME METABOLISM	0.07	0.24	0.89
HALLMARK PROTEIN SECRETION	0.08	0.22	0.90
HALLMARK INTERFERON GAMMA RESPONSE	0.08	0.20	0.90
HALLMARK ESTROGEN RESPONSE LATE	0.13	0.25	0.96
HALLMARK INTERFERON ALPHA RESPONSE	0.17	0.29	0.99
HALLMARK ALLOGRAFT REJECTION	0.19	0.30	0.99
HALLMARK APICAL SURFACE	0.29	0.33	1.00
HALLMARK KRAS SIGNALING DN	0.28	0.37	1.00
HALLMARK UV RESPONSE DN	0.33	0.43	1.00
HALLMARK KRAS SIGNALING UP	0.47	0.55	1.00
HALLMARK SPERMATOGENESIS	0.74	0.91	1.00
HALLMARK FATTY ACID METABOLISM	0.96	0.99	1.00
HALLMARK PEROXISOME	0.99	0.99	1.00

**Table 3:** Upregulated gene sets after 2h OGD.

NAME	NOM p-val	FDR q-val	FWER p-val
HALLMARK TNFA SIGNALING VIA NFKB	0.00	0.00	0.00
HALLMARK HYPOXIA	0.00	0.00	0.00
HALLMARK INFLAMMATORY RESPONSE	0.00	0.00	0.00
HALLMARK EPITHELIAL MESENCHYMAL TRANSITION	0.00	0.00	0.00
HALLMARK KRAS SIGNALING UP	0.00	0.00	0.00
HALLMARK IL2 STAT5 SIGNALING	0.00	0.00	0.00
HALLMARK COMPLEMENT	0.00	0.00	0.01
HALLMARK IL6 JAK STAT3 SIGNALING	0.01	0.00	0.02
HALLMARK UV RESPONSE DN	0.00	0.01	0.03
HALLMARK ALLOGRAFT REJECTION	0.00	0.01	0.05
HALLMARK COAGULATION	0.00	0.01	0.06
HALLMARK APOPTOSIS	0.00	0.03	0.15
HALLMARK ANDROGEN RESPONSE	0.01	0.05	0.30
HALLMARK MYC TARGETS V2	0.04	0.05	0.33
HALLMARK GLYCOLYSIS	0.02	0.07	0.41
HALLMARK UV RESPONSE UP	0.06	0.13	0.65
HALLMARK ADIPOGENESIS	0.03	0.12	0.66
HALLMARK MTORC1 SIGNALING	0.02	0.12	0.68
HALLMARK P53 PATHWAY	0.04	0.12	0.69
HALLMARK KRAS SIGNALING DN	0.11	0.14	0.76

**Table 4:** Upregulated gene sets after 6h hypoxia.

NAME	NOM p-val	FDR q-val	FWER p-val
HALLMARK HYPOXIA	0.00	0.00	0.00
HALLMARK GLYCOLYSIS	0.00	0.00	0.00
HALLMARK MITOTIC SPINDLE	0.00	0.01	0.02
HALLMARK G2M CHECKPOINT	0.00	0.02	0.09
HALLMARK PI3K AKT MTOR SIGNALING	0.01	0.07	0.33
HALLMARK APICAL JUNCTION	0.02	0.10	0.49
HALLMARK MTORC1 SIGNALING	0.01	0.10	0.54
HALLMARK P53 PATHWAY	0.04	0.14	0.73
HALLMARK IL2 STAT5 SIGNALING	0.04	0.14	0.75
HALLMARK E2F TARGETS	0.04	0.15	0.81
HALLMARK MYOGENESIS	0.07	0.15	0.84
HALLMARK ANGIOGENESIS	0.23	0.29	0.99
HALLMARK ESTROGEN RESPONSE EARLY	0.15	0.32	0.99
HALLMARK HEME METABOLISM	0.26	0.46	1.00
HALLMARK SPERMATOGENESIS	0.33	0.47	1.00
HALLMARK ADIPOGENESIS	0.30	0.48	1.00
HALLMARK NOTCH SIGNALING	0.42	0.55	1.00
HALLMARK MYC TARGETS V2	0.51	0.71	1.00
HALLMARK ESTROGEN RESPONSE LATE	0.55	0.69	1.00
HALLMARK DNA REPAIR	0.69	0.80	1.00

**Table 5:** Upregulated gene sets after 6h no glucose.

NAME	NOM p-val	FDR q-val	FWER p-val
HALLMARK EPITHELIAL MESENCHYMAL TRANSITION	0.00	0.00	0.00
HALLMARK MYC TARGETS V2	0.00	0.00	0.00
HALLMARK UNFOLDED PROTEIN RESPONSE	0.00	0.00	0.00
HALLMARK TNFA SIGNALING VIA NFKB	0.00	0.00	0.00
HALLMARK INFLAMMATORY RESPONSE	0.00	0.01	0.03
HALLMARK IL2 STAT5 SIGNALING	0.00	0.01	0.06
HALLMARK MTORC1 SIGNALING	0.00	0.02	0.08
HALLMARK WNT BETA CATENIN SIGNALING	0.01	0.02	0.10
HALLMARK ALLOGRAFT REJECTION	0.01	0.03	0.16
HALLMARK P53 PATHWAY	0.00	0.05	0.32
HALLMARK KRAS SIGNALING DN	0.05	0.07	0.47
HALLMARK MYOGENESIS	0.05	0.12	0.66
HALLMARK HYPOXIA	0.05	0.18	0.84
HALLMARK KRAS SIGNALING UP	0.11	0.19	0.89
HALLMARK UV RESPONSE DN	0.12	0.27	0.97
HALLMARK CHOLESTEROL HOMEOSTASIS	0.17	0.26	0.97
HALLMARK ANDROGEN RESPONSE	0.17	0.28	0.98
HALLMARK PI3K AKT MTOR SIGNALING	0.18	0.28	0.99
HALLMARK APICAL JUNCTION	0.15	0.28	0.99
HALLMARK COMPLEMENT	0.19	0.31	0.99

**Table 6:** Upregulated gene sets after 6 h of OGD.

NAME	NOM p-val	FDR q-val	FWER p-val
HALLMARK HYPOXIA	0.00	0.00	0.00
HALLMARK EPITHELIAL MESENCHYMAL TRANSITION	0.00	0.00	0.00
HALLMARK TNFA SIGNALING VIA NFKB	0.00	0.00	0.00
HALLMARK INFLAMMATORY RESPONSE	0.00	0.00	0.00
HALLMARK IL2 STAT5 SIGNALING	0.00	0.00	0.00
HALLMARK UV RESPONSE DN	0.00	0.00	0.00
HALLMARK KRAS SIGNALING UP	0.00	0.00	0.00
HALLMARK MTORC1 SIGNALING	0.00	0.00	0.00
HALLMARK UNFOLDED PROTEIN RESPONSE	0.00	0.00	0.01
HALLMARK GLYCOLYSIS	0.00	0.00	0.02
HALLMARK ALLOGRAFT REJECTION	0.00	0.01	0.05
HALLMARK ANDROGEN RESPONSE	0.01	0.02	0.16
HALLMARK WNT BETA CATENIN SIGNALING	0.02	0.02	0.17
HALLMARK P53 PATHWAY	0.00	0.03	0.28
HALLMARK MYC TARGETS V1	0.01	0.04	0.35
HALLMARK COMPLEMENT	0.02	0.05	0.45
HALLMARK APOPTOSIS	0.02	0.07	0.54
HALLMARK MYC TARGETS V2	0.06	0.07	0.58
HALLMARK APICAL JUNCTION	0.03	0.08	0.64
HALLMARK PI3K AKT MTOR SIGNALING	0.08	0.10	0.75

### Gene regulation is dominated by c-JUN, NFκB and STAT3 after 2hs of hypoxia or OGD

We further examined the GSEA analysis for transcription factors active in hypoxia or OGD. To this end, we chose to download chromatin-immuno-precipitation sequencing (ChIP-seq) data for c-JUN, NF-κB, STAT3, and HIF1α (JNSH).

**Fig 2:**
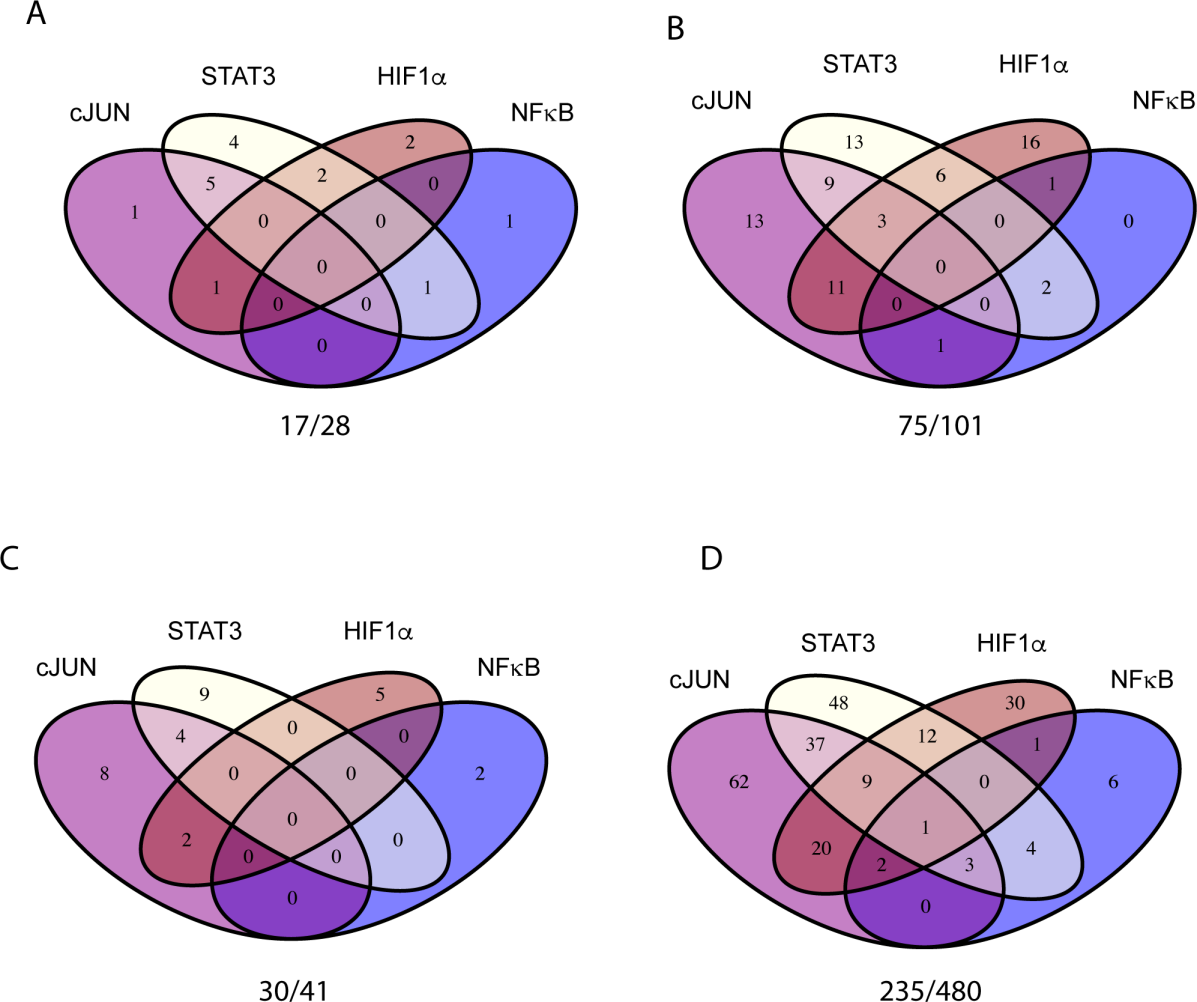
HIF1α is not the primary early transcription factor regulating gene expression in hypoxic or OGD–treated pericytes. ChIP-seq data for c-JUN, NFκB, STAT3 or HIF1α were analysed for binding peaks near (5Kbp) the transcriptional binding site of genes regulated (fc>1.5, p<0.05) in hypoxic or OGD treated pericytes. Venn diagrams are for (A) 2h hypoxia, (B) 6h hypoxia, (C) 2h OGD or (D) 6h OGD-treated pericytes. The numbers of genes regulated by either of the four transcription factors (x) compared to the total number of regulated genes (y) are presented as (x/y) below each graph.

When comparing the hypoxic 2h stimulation with the OGD condition we found that 28 genes were upregulated by the hypoxia at fc >1.5, p<0.05 whereas 41 genes were regulated by OGD (Fig 1C and 1D). In the JNSH combination of transcription factors, we identified 17/28 bound regulated genes (BRGs) in the hypoxic condition (61%) (Fig 2A). In the 2h OGD treatment there were 30/41 BRGs (73%) (Fig 2C).

Many of the genes were targeted by two or more of the JNSH transcription factor combinations. The 2h hypoxia treatment induced 17 BRGs, out of which only one was unique for the c-JUN transcription factor. NF-κB induced two BRGs, out of which one was unique for NF-κB and the other one, IL6, also was bound by STAT3. Apart from IL6, STAT3 regulated 11 genes. In total 4 genes were unique for STAT3. HIF1α regulated 2 unique genes of in total 5 BRGs at 2h, suggesting that c-JUN, NFκB and STAT3 substantiated the early responses to hypoxia.

Two h OGD treatment lead to 14 c-JUN BRGs out of which 8 were unique for c-JUN, 4 shared with STAT3 and 2 with HIF1α. 13 STAT3 OGD BRGs were distributed to STAT3 out of which 9 were unique, and 4 are shared between STAT3 and c-JUN. HIF1α regulated 5 unique BRGs of a total of 7. NFκB regulated a total of 2 unique BRGs. This suggested to us that HIF1α was not the dominant transcription factor regulating early OGD or hypoxic responses of pericytes.

### HIF1α activity is stronger after 6 hs than after 2hs of hypoxia or OGD

After 6h of hypoxic stimulation a total of 75 BRGs were regulated by the JNSH transcription factor combination. That was in comparison to a total of 101 genes that are regulated fc >1.5, p <0.05 at 6h of hypoxia. C-JUN regulated 37 BRGs at 6 h of stimulation, out of which 13 were unique for c-JUN. The majority of BRGs were shared with STAT3 and/or HIF1α at 6h (Fig 2B). 4 BRGs were associated with NFκB at the 6-h stimulation; IL6 was still a shared BRG with STAT3 at this time point, albeit down regulated. STAT3 regulated 13 unique BRGs at 6 h of hypoxia, whereas 9 BRGs were shared with c-JUN and 6 with HIF1α. 2 STAT3 BRGs were shared with NFκB at 6h of hypoxia. Among the JNSH TFs, HIF1α regulated the highest quantity of unique genes (16), suggesting that the HIF1α targeted expression of genes took longer time than either of the transcription factors c-JUN, STAT3 or NFκB, but that HIF1α dominated the treatment effects at 6h of hypoxia. HIF1α shared 11 BRGs with c-JUN, 3 with STAT3/c-JUN, 6 with STAT3 and 1 with NFκB, respectively (Fig 2B).

After 6 h of OGD treatment JNSH transcription factors have 235 BRGs out of a total of 480 genes regulated at a fc > 1.5, p <0.05. Among the 235 BRGs, 134 were regulated by c-JUN. 62 of these genes were unique to c-JUN, 37 shared with STAT3, 20 with HIF1α. Notably 9 genes were BRGs of c-JUN, NFκB and STAT3. Out of 114 total BRGs, STAT3 had 48 unique BRGs in the OGD treatment. 12 BRGs were shared with HIF1α and 4 with NFκB (Fig 2D). HIF1α had a total of 75 BRGs in the OGD condition; approximately 56% of the amount of c-JUN targets and 66% of the STAT3 targets (Fig 2D). This was in comparison to the hypoxic treatment, where the amount of BRGs after 6h treatment was almost equal between HIF1α (37 BRGs) and the c-JUN (37 BRGs) or STAT3 (33 BRGs). Out of the total 75 BRGs in the OGD condition, 30 BRGs were unique to HIF1α, the others were shared with STAT3 (12 BRGs), c-JUN (20 BRGs), NFκB (1 BRG) or with combinations of c-JUN/STAT3, c-JUN/NFκB or c-JUN/NFκB/STAT3 transcription factors. For NFκB, 17 genes were regulated by the OGD treatment (Fig 2D), which was an increase compared to the 4 BRGs regulated after 6h of hypoxic treatment (Fig 2B). 6 BRGs are uniquely regulated by NFκB at 6h after OGD treatment. As mentioned, 4 are shared with STAT3 and 1 with HIF1α; the other BRGs were shared with combinations of the c-JUN, NFκB or STAT3 transcription factors.

This suggests, that HIF1α does not initiate the hypoxic response of brain pericytes at 2h of treatment, and that HIF1α is not the major initiator or contributor to their response to OGD at 2h or 6h of *in vitro* treatment.

### STAT3 is an early regulator of hypoxia and OGD in pericytes

**Fig 3:**
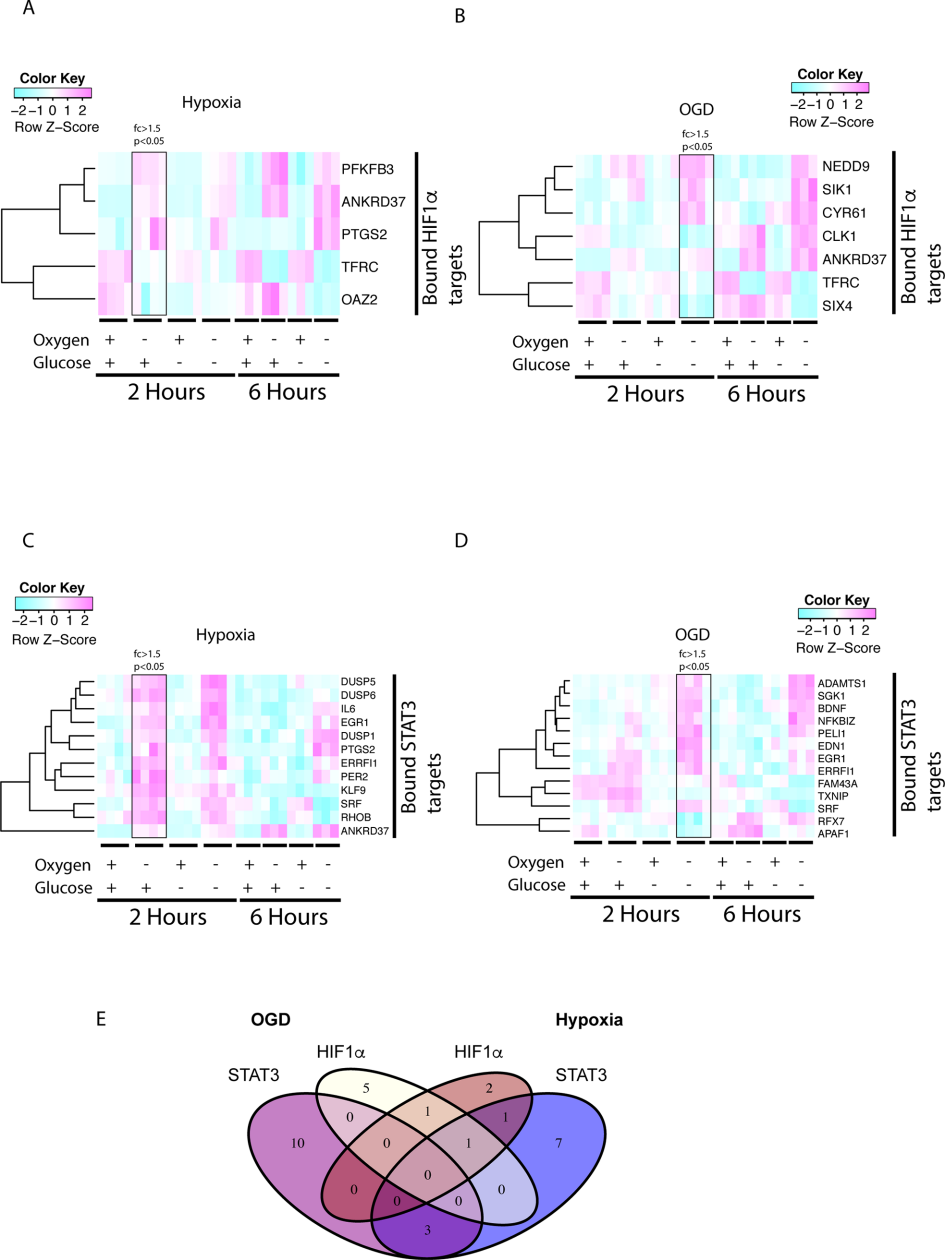
STAT3 drives both hypoxic and OGD responses stronger than HIF1α in 2h treated pericytes. Bound regulated genes (BRGs) with transcription factor occupancy within 5Kbp of a TSS for STAT3 and HIF1α were identified in by comparing ChIP-seq data to the pericytic Illumina gene expression data. (A) HIF1α alpha BRGs in the 2h hypoxic condition (fc>1.5, p<0.05) was plotted in a heat map. Panel (B) shows HIF1α BRGs in the OGD condition plotted in a heat map. (C) STAT3 BRGs after 2h of hypoxia and (D) 2h OGD. (E) A Venn diagram was constructed to highlight the differences in STAT3 or HIF1α BRG distribution between 2h hypoxic or OGD conditions.

STAT3, and IL6, have in a number of in vivo studies been suggested to precondition better recovery after stroke in mouse models [17,18,41]. In order to specifically delineate when STAT3-regulated genes start to be expressed in pericytes in the hypoxic and OGD treatments, we identified genes with an fc>1.5, p <0.05 bound by STAT3 or HIF1α after 2 or 6h hypoxia or OGD. Fig 3A shows the five genes that are BRGs of HIFα after hypoxic treatment of pericytes. There were 7 HIF1α BRGs after 2h ODG treatment (Fig 3B). Out of 7 HIF1α regulated BRGs in OGD, 1 gene was shared with between OGD and hypoxia and 2 BRGs were regulated in the hypoxic condition (Fig 3E). In Fig 3C, STAT3 hypoxic BRGs are depicted, note that IL6 is a target of STAT3 and that it is upregulated upon 2h hypoxia, OGD and 6h OGD but not after 6h hypoxia. At 2h after OGD treatment 13 genes were regulated as STAT3 BRGs (Fig 3D) out which 3 were shared with the hypoxic treatment (Fig 3E). 7 genes were unique for the hypoxic treatment (Fig 3E).

**Fig 4:**
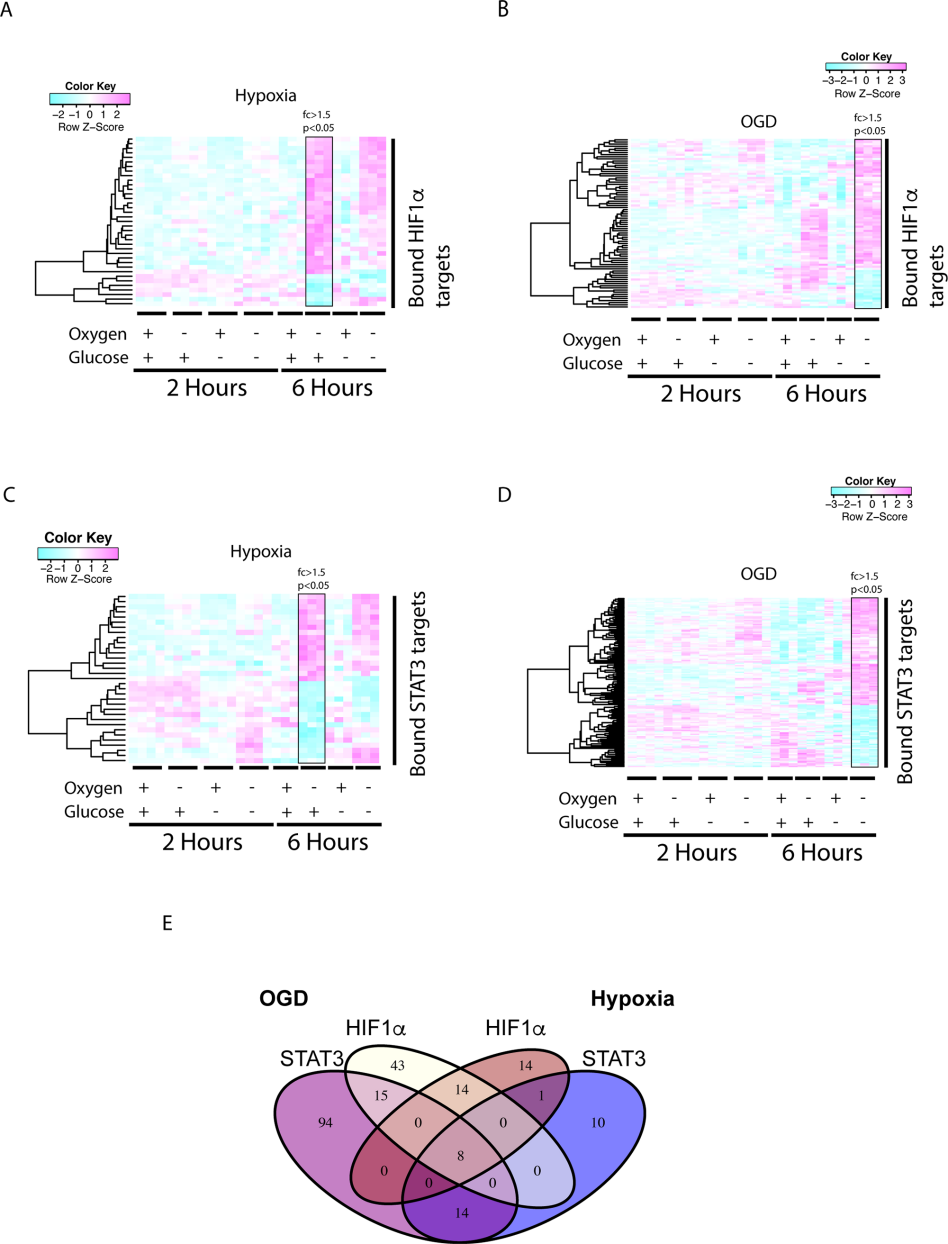
STAT3 dominates the OGD responses but shows equal activity to HIF1α in the hypoxic responses in 6h treated pericytes. STAT3 or HIF1α bound regulated genes (BRGs) with binding site(s) 5Kbp of a TSS were identified by comparing ChIP-seq data to the pericytic Illumina gene expression data. (A) HIF1α alpha BRGs in the 6h hypoxia or (B) OGD condition (fc>1.5, p <0.05) was plotted in a heat map. Panel (C) shows STAT3 BRGs in the hypoxia and (D) OGD condition plotted in a heat map. (E) A Venn diagram was constructed to highlight the differences in STAT3 or HIF1α BRG distribution between 6 h hypoxic and OGD conditions.

The 6h hypoxic treatment leads to the induction of 37 HIF1α BRGs compared to the regulation of 33 STAT3 BRGs (Fig 4A, 4C and 4E).

After 6h of OGD treatment the HIF1α BRGs increased to 80 (Fig 4B), whereas there were 131 STAT3 BRGs (Fig 4D). Lists of the regulated genes can be found in the S1 File. There are 15 shared STAT3/HIF1α BRGs. If one compares the hypoxic to OGD conditions for STAT3 BRGs, 10 genes were exclusive BRGs for hypoxia, 14 shared with OGD and 94 specific for the OGD treatment (Fig 4E). The HIF1α response to hypoxia induces 14 BRGs, 14 BRGs shared with OGD and 43 genes were regulated upon OGD only (Fig 4E) indicating that STAT3 regulated the early transcriptional responses to hypoxia and OGD.

### Validation of Illumina data with qPCR

**Fig 5:**
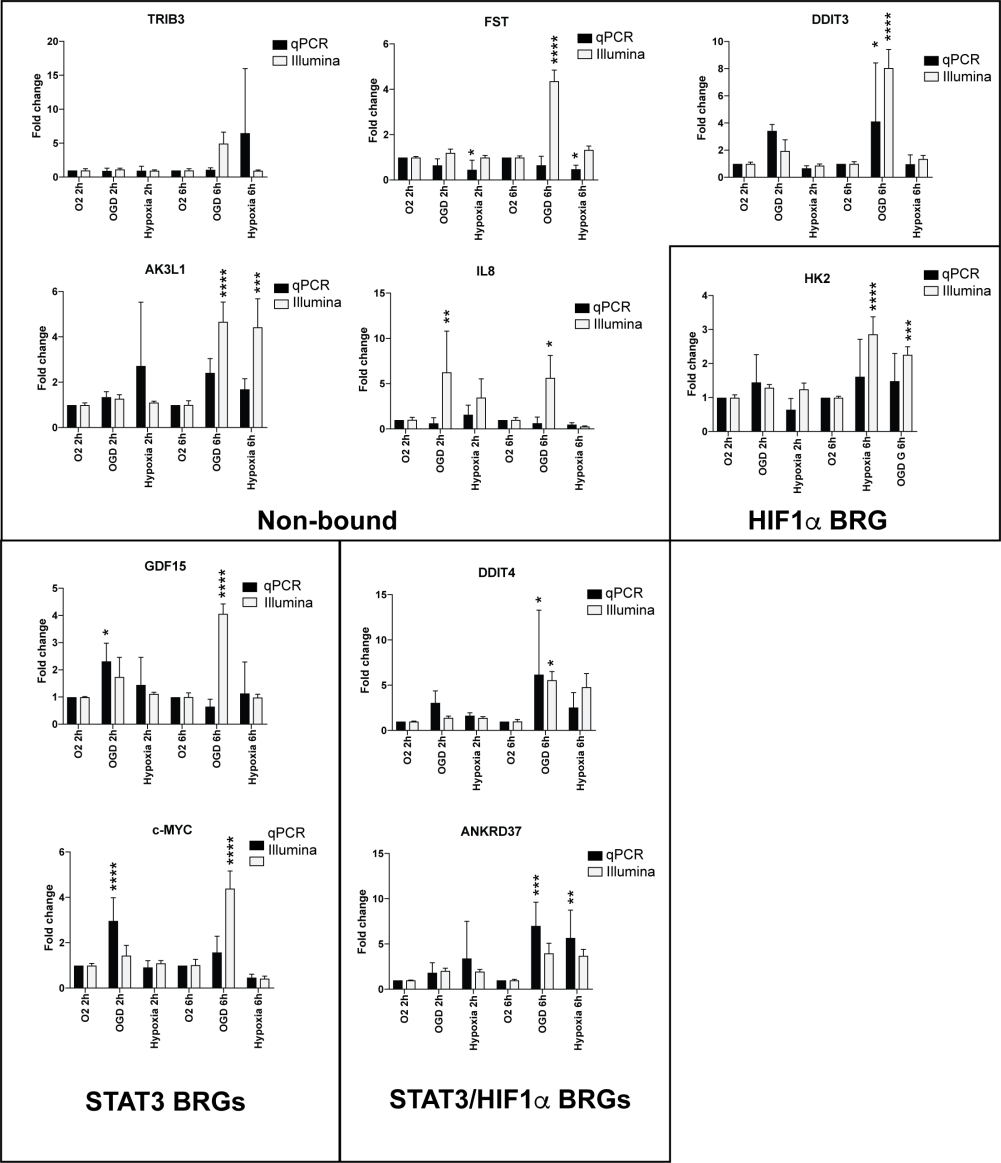
STAT3 BRGs c-MYC and GDF are upregulated 2h after treatment. QPCR of normoxic, hypoxia or OGD treated SVZ pericytes with 2h or 6h treatments. SVZ pericytes were analysed for differences in expression of the indicated transcripts with qPCR (n = 3) and compared to Illumina data (n = 4 or n = 3). The individual analysed transcripts were grouped into non-bound (not bound by either STAT3 or HIF1α as BRGs), STAT3 BRGs, HIF1α/STAT3 combined BRGs or HIF1α BRGs. Statistic analysis was made using 2-way ANOVA and error bars represent standard deviations of the mean (SEM); *p<0.05, ** P<0.01, ***p<0.001, ****p<0.0001.

Next we performed qPCR to validate observed gene changes. QPCR was performed using human brain pericytes treated with normoxia, OGD or hypoxia for 2 or 6h, respectively. We could detect differences in the transcriptional level on STAT3 BRGs c-MYC and GDF15 that were significant at 2h of OGD (Fig 5). This response was faster than observed using the Illumina analysis, where the expression of c-MYC and GDF15 was significantly higher after 6h of OGD (Fig 5). The combined STAT3/HIF1α BRGs responded slower and were significantly upregulated after 6h, but not after 2h of OGD. HK2, a HIF1α BRG failed to be significantly upregulated both at 2h and 6h treatments. This strengthens the role for STAT3 as the initiating factor for both a broad transcriptional response and in particular, the regulation of important angiogenic genes c-MYC [42] and GDF15 [43].

### Phosphorylated STAT3 protein is upregulated before HIF1α in human brain pericytes

Since both STAT3 and HIF1α require nuclear localisation to exert their transcriptional activities, we used confocal imaging to measure nuclear localisation and expression of pS727-STAT3 and HIF1α. PS727-STAT3 was chosen because it previously was shown that phosphorylation of STAT3 at S727 mediated transcriptional activity to the STAT3 protein [44].

**Fig 6:**
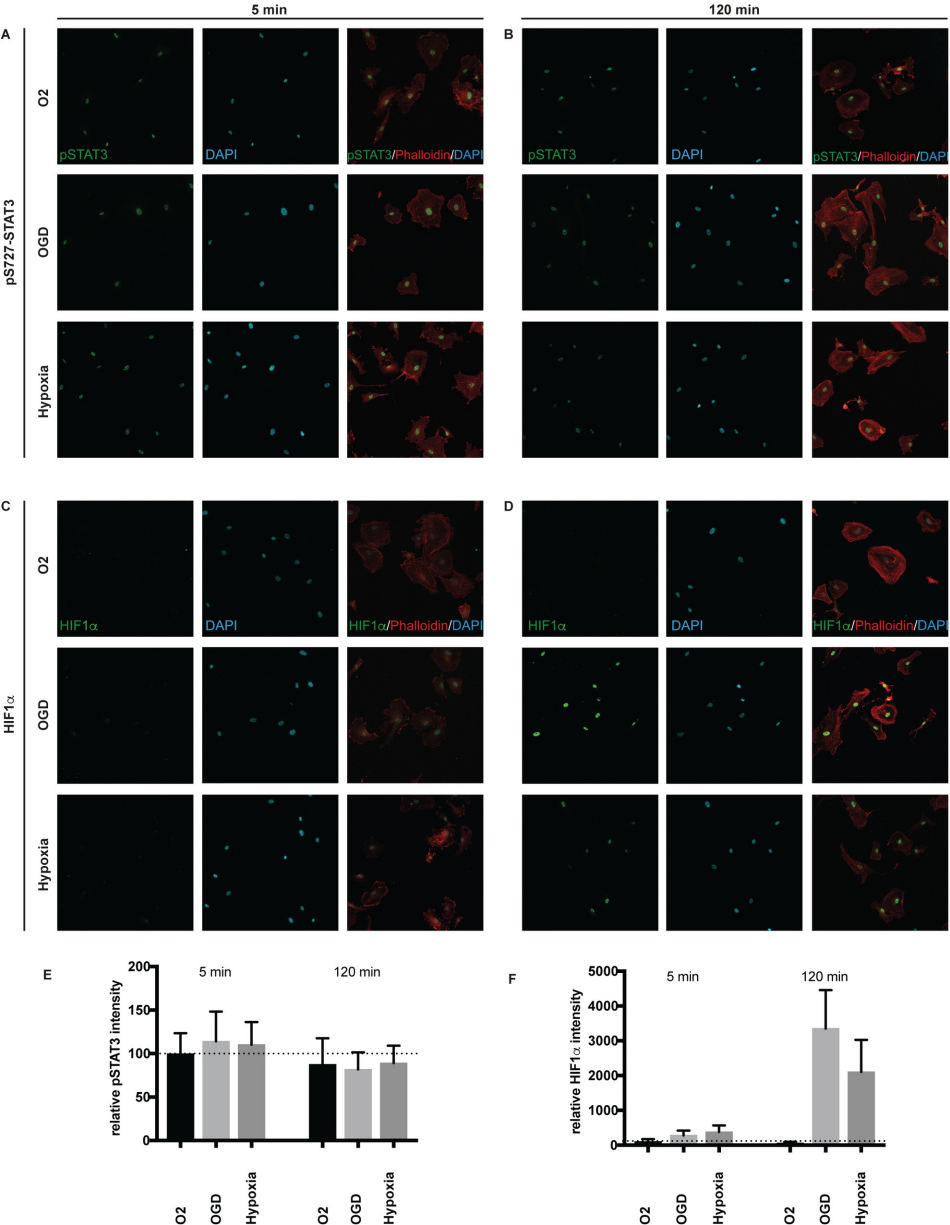
PSTAT3 is increased in the nucleus of pericytes after 5min of OGD or hypoxia. Localization of pSTAT3 and HIF1a over time. (A) Confocal images showing pS727STAT3 (green), DAPI (blue) and Phalloidin (red) after 5 minute treatment of Normoxia (upper panel), OGD (middle panel) and OGD + Glucose (lower panel). (B) Confocal images showing pS727STAT3 (green), DAPI (blue) and Phalloidin (red) after 120 minute treatment of normoxia (upper panel), OGD (middle panel) and hypoxia (lower panel). (C) Confocal images showing HIF1α (green), DAPI (blue) and Phalloidin (red) after 5 minute treatment of Normoxia (upper panel), OGD (middle panel) and hypoxia (lower panel). (D) Confocal images showing HIF1α (green), DAPI (blue) and Phalloidin (red) after 120 minute treatment of Normoxia (upper panel), OGD (middle panel) and hypoxia (lower panel). (E) Quantification of nuclear intensity of pS727STAT3 in all conditions at 5 and 120 min. (F) Quantification of nuclear intensity of pS727STAT3 in all conditions at 5 and 120 min.

For this purpose, pericytes were cultured on cover slips, treated with normoxia, hypoxia or OGD and stained for pS727-STAT3 or HIF1α. Unexpectedly, pericytes expressed pS727-STAT3 at steady state normoxic conditions (Fig 6A). Upon 5 min OGD or hypoxia, there was however a slight increase of pS727-STAT3. The increased STAT3 phosphorylation was not detected at 120min, neither with OGD or hypoxic treatment. HIF1α was only slightly detectable at 5 min of OGD or hypoxia, but dramatically increased after 120min OGD or hypoxic treatment (Fig 6). In order to further measure the levels of phosphorylated STAT3 compared to HIF1α, we performed a series of western blots. The temporal design was chosen based on the activity of STAT3 and HIF1α in the immunocytochemistry. We exposed brain pericytes (ScienCell) for 5, 10, 15 min with normoxia, hypoxia or OGD and isolated nuclear extracts to decipher if the hypoxic and OGD responses of early STAT3 activation prior to HIF1α can be confirmed also in another brain pericyte line. The western blot analysis corroborated that nonstimulated cells contain low levels of activated pS727- pY705-STAT3 but non-detectable levels of HIF1α. Rapidly, after only 5min stimulation with OGD, STAT3 became phosphorylated at both the transcriptionally promoting pS727- and the dimerisation inducing pY705-residues (S1A Fig). At 10min, the phosphorylation was diminished and returned to background levels at 15min (S1A Fig). For HIF1α, stabilisation of the protein could be slightly detected after 5min of OGD, and increased at 10 and 15 min. Under hypoxic conditions, HIF1α activity was only first detected after 10min hypoxia. Since the activity of both HIF1α and STAT3 occurs primarily in the nucleus in pericytes (Fig 6 and (S1A Fig), we examined if there was any additional accumulation of HIF1α in whole cell extracts of pericytes. For the time points studied, the HIF1α protein levels were highest at the 120min time point, suggesting that full activity of HIF1α requires long and steady hypoxia or OGD to mediate maximum HIF1α stabilisation (S1 Fig). This suggested that the protein phosphorylation of STAT3 early mediated the transcriptional responses of pericytes to hypoxia or OGD.

### Analysis of STAT3 and HIF1α BRGs by GO:Term analysis

Next we asked which cellular processes might be affected by the early STAT3 activation. We imported STAT3 or HIF1α BRGs into the Panther GO analysis software. The predominant STAT3 enriched GO:terms indicated that the responses mediated by STAT3 were shaping the cellular response to OGD or hypoxia by the alteration of metabolism and angiogenesis at 6h, but also at 2h hypoxia treatment (Tables 7–9). For HIF1α, there were no significantly upregulated GO:term pathways in the Panther analysis at 2h treatment. At 6h treatment, however, HIF1α BRG metabolic and angiogenic GO:terms were significantly enriched for (Tables 10 and 11). This showed that genes that are regulated early in pericytes in response to OGD or hypoxia are important for the angiogenic, apoptotic and metabolic control and are under the regulation of STAT3.

**Table 7:** STAT3 BRGs 6h hypoxia.

GO biological process complete	Fold Enrichment	P-value
positive regulation of cytokine production (GO:0001819)	12.44	2.07E-07
regulation of cell death (GO:0010941)	4.94	7.28E-07
regulation of cell adhesion (GO:0030155)	8.3	9.14E-07
regulation of apoptotic process (GO:0042981)	4.96	2.16E-06
positive regulation of multicellular organismal process (GO:0051240)	4.91	2.40E-06
regulation of programmed cell death (GO:0043067)	4.91	2.40E-06
regulation of angiogenesis (GO:0045765)	15.03	2.86E-06
positive regulation of angiogenesis (GO:0045766)	21.95	3.52E-06
regulation of vasculature development (GO:1901342)	13.75	4.72E-06
regulation of cytokine production (GO:0001817)	7.95	5.63E-06

**Table 8:** STAT3 BRGs 6h OGD.

GO biological process complete	Fold Enrichment	P-value
response to organic substance (GO:0010033)	3.2	2.11E-16
regulation of metabolic process (GO:0019222)	2.04	1.25E-14
negative regulation of biological process (GO:0048519)	2.3	4.46E-14
regulation of programmed cell death (GO:0043067)	3.98	1.34E-13
regulation of cellular metabolic process (GO:0031323)	2.05	2.53E-13
regulation of cell death (GO:0010941)	3.79	2.62E-13
response to organic cyclic compound (GO:0014070)	5.22	3.23E-13
response to chemical (GO:0042221)	2.43	3.88E-13
regulation of apoptotic process (GO:0042981)	3.91	5.06E-13
positive regulation of biological process (GO:0048518)	2.12	7.55E-13

**Table 9:** STAT3 BRGs 2h Hypoxia.

GO biological process complete	Fold Enrichment	P-value
response to drug (GO:0042493)	15.4	5.42E-09
cellular response to drug (GO:0035690)	34.05	9.16E-09
response to glucocorticoid (GO:0051384)	62.18	1.14E-08
response to corticosteroid (GO:0031960)	55.84	1.92E-08
negative regulation of cell communication (GO:0010648)	11.02	7.30E-08
negative regulation of signaling (GO:0023057)	10.99	7.48E-08
response to hormone (GO:0009725)	14	1.48E-07
response to organic cyclic compound (GO:0014070)	13.96	1.50E-07
response to toxic substance (GO:0009636)	21	1.55E-07
cellular response to oxygen-containing compound (GO:1901701)	13.56	1.83E-07

**Table 10:** HIF1α BRGs 6h hypoxia.

GO biological process complete	Fold Enrichment	P-value
cellular carbohydrate metabolic process (GO:0044262)	22.15	3.39E-07
monosaccharide metabolic process (GO:0005996)	16.78	1.63E-06
glycolytic process through fructose-6-phosphate (GO:0061615)	63.89	1.91E-05
carbohydrate metabolic process (GO:0005975)	8.21	2.05E-05
canonical glycolysis (GO:0061621)	66.45	1.72E-05
glycolytic process through glucose-6-phosphate (GO:0061620)	63.89	1.91E-05
glucose catabolic process to pyruvate (GO:0061718)	66.45	1.72E-05
fructose metabolic process (GO:0006000)	> 100	4.33E-06
anatomical structure development (GO:0048856)	2.37	1.17E-05
hexose metabolic process (GO:0019318)	17.63	1.03E-05

**Table 11:** HIF1α BRGs 6h OGD.

GO biological process complete	Fold Enrichment	P-value
response to hypoxia (GO:0001666)	11.49	5.15E-12
response to decreased oxygen levels (GO:0036293)	11.23	7.07E-12
response to oxygen levels (GO:0070482)	10.5	1.76E-11
response to abiotic stimulus (GO:0009628)	4.41	4.18E-08
regulation of angiogenesis (GO:0045765)	10.52	5.25E-08
response to stress (GO:0006950)	2.55	9.35E-08
cellular response to stress (GO:0033554)	3.57	1.16E-07
regulation of vasculature development (GO:1901342)	9.62	1.17E-07
regulation of cell death (GO:0010941)	3.35	7.00E-07
cellular response to hypoxia (GO:0071456)	11.17	7.95E-07

## Discussion

In stroke, the lack of oxygen (hypoxia) is often accompanied by the lack of glucose due to deficient blood flow. Here we investigated the effect hypoxia, glucose or the combination of oxygen and glucose deprivation (OGD) has on the transcriptome of human brain pericytes as one of the early responders in stroke and key regulators in the neurovascular unit. HIF1α has been suggested to be the master regulator of hypoxia. HIF1α protein is stabilised by the hypoxic condition [14,15]. 2hs of hypoxia leads to maximum protein levels in kinetics of HIF1α stabilisation in HeLa cells [45]. The HIF1α stabilization and increased HIF1α protein levels can be detected as early as after 15 min after hypoxia in HeLa cells [45]. It is however not clear if HIF1α is the predominant factor regulating transcription in hypoxia since many other transcription factors are induced by the cellular stress that hypoxia confers.

Here we show that STAT3 precedes HIF1α in regulating STAT3 prototypic target genes in 2h hypoxic pericytes, compared to HIF1α regulation of HIF1α BRGs. In addition, the transcription factors c-JUN and NFκB contribute to HIF1α independent transcriptional responses early in the hypoxic gene expression programme. For the 2h OGD condition the STAT3 dominance is even clearer. STAT3 regulates 1.9-fold more genes than HIF1α at 2h, and 1.5-fold more genes than HIF1α at 6h of OGD, suggesting that HIF1α is activating its target genes at a later time point than STAT3.

Hypoxic treatment induces IL6 mRNA at 2hs after treatment, whereas after 6 hs IL6 is downregulated. OGD induces IL6 mRNA after 2 and 6 hs. This suggests that the STAT3 effect differs between hypoxia and OGD conditions. IL6 has previously been shown to be important for the vascularisation and recovery after stroke [17,18,41] and treatment experiments have shown that IL6 can be used to reduce infarct volumes in a reperfusion mouse model (MCAO) of stroke [17]. Consistently, IL6^-/-^ mice have increased stroke infarct volumes [18]. In a study on endothelial cells conditionally lacking STAT3, the major transcription factor regulated by IL6, increased infarct volumes and reduced neo-vascularisation leads to impaired motor function in the mice after stroke [41]. In the same study it was shown that the vasculature of the stroke mice changed to a non-capillary large vessel phenotype in the STAT3 endothelial knockout stroke mice, suggesting that IL6 production of nearby cells, for example pericytes, would contribute to tissue recovery after stroke [41]. In pericytes, IL6 is expressed under non-stimulated conditions, but increases upon hypoxia or OGD treatment. Interestingly, STAT3 and NFκB can bind the IL6 promoter [46] [47]. Since IL6 activates STAT3, which then can activate IL6, it is unlikely that STAT3 is initiating IL6 transcription. NFκB has been shown to activate the IL6 promoter in astrocytes in an NFκB DNA binding site dependent manner [47]. We show that STAT3 was activated rapidly, as early as after 5min, upon OGD treatment of two pericyte cell lines, respectively. Activation-induced phosphorylation of STAT3 confers dimerisation, DNA binding and transcription of target genes. We show that nuclear expression of pS727-STAT in human brain pericytes increased after 5 min of OGD or hypoxia. The mechanism underlying the very early regulation of pSTAT3 needs to be deciphered, but it is likely that de novo synthesis of IL6 is not required and that growth factors activating ERK1/2 might be part of the pS727-STAT3 cascade as described [48]. In contrast, HIF1α was at most expressed and stabilised after 120 min OGD and hypoxic treatment and very little HIF1α was detected after 5 min treatments. The temporal regulation of hypoxia inducible genes 2h after OGD is dominated by STAT3 BRGs both in the Illumina gene expression data and in qPCR validations of the Illumina data. More so, the qPCR data showed that the STAT3 BRGs c-MYC and GDF15 are significantly upregulated already after 2h of OGD, whereas in the Illumina data, c-MYC and GDF15 are significantly upregulated first after 6h. BRGs co-regulated by STAT3 and HIF1α were validated to be significantly expressed first after 6h of OGD treatment, suggesting that the collaborative action of STAT3 and HIF1α mediates transcriptional activation after that of STAT3 alone. The HIF1α BRG HK2 was not significantly induced, neither at the 2h or 6h OGD or hypoxia treatments, respectively.

We conclude that STAT3 phosphorylation activity, that mediates transcriptional activation of STAT3, was present at its maximum already after 5min of OGD treatment. The transcription mediated significant overexpression of the STAT3 BRGs c-MYC and GDF15 after 2h of OGD treatment suggesting that the protein modifications of STAT3 results in early activation of STAT3 target genes that directs the early hypoxic responses. GO:term analysis suggests that the major STAT3 BRG responses regulate metabolic and angiogenic processes. This implies that STAT3 initiates a transcriptional program necessary for pericytes to adapt to hypoxic and OGD conditions prior to the time point when HIF1α’s activity is induced.

Our findings suggest that STAT3 is a potential target for intervention in pathologies where there is hypoxia or OGD, e.g. asphyxiation, tumor pathologies or in ischemic stroke.

## Supporting information

S1 Fig PSTAT3 reaches its highest levels at 5min, HIF1α at 120min of ODG. Western blot of (A) nuclear extracts (B) or whole cell extracts from Sciencell pericytes treated with normoxia, hypoxia or OGD. (A) 5,10 or 15 min treatments followed by extraction of nuclear proteins. In (A) anti-pS727STAT3 was used to visualise nuclear phosphorylation of STAT3 required for transcriptional activity. PY705 was used to measure the nuclear phosphorylation required for dimerisation and nuclear translocation of STAT3. Nuclear anti-HIF1α was measured at 5, 10 or 15 minutes of treatments. Anti-STAT3 indicated the steady state nonphosphorylated STAT3 in the nuclear extracts and was compared to the pS727- and pY705-STAT3 levels. (B) Pericytes treated with normoxia, hypoxia or OGD for 15, 45, 75 or 120min prior to whole cell lysis. Western blot of pY705-STAT3, HIF1α or total STAT3.
(TIF)

S1 File Raw data from Figs 3–6. (XLSX)

## References

[1] Hawkins BT, Davis TP (2005) The blood-brain barrier/neurovascular unit in health and disease. Pharmacol Rev 57: 173–185. doi: 10.1124/pr.57.2.4 15914466 10.1124/pr.57.2.4

[2] Sweeney MD, Ayyadurai S, Zlokovic BV (2016) Pericytes of the neurovascular unit: key functions and signaling pathways. Nat Neurosci 19: 771–783. doi: 10.1038/nn.4288 27227366 10.1038/nn.4288 PMC5745011

[3] Bell RD, Winkler EA, Sagare AP, Singh I, LaRue B, Deane R, et al (2010) Pericytes control key neurovascular functions and neuronal phenotype in the adult brain and during brain aging. Neuron 68: 409–427. doi: 10.1016/j.neuron.2010.09.043 21040844 10.1016/j.neuron.2010.09.043 PMC3056408

[4] Winkler EA, Bell RD, Zlokovic BV (2011) Lack of Smad or Notch leads to a fatal game of brain pericyte hopscotch. Dev Cell 20: 279–280. doi: 10.1016/j.devcel.2011.03.002 21397835 10.1016/j.devcel.2011.03.002

[5] Beckman JD, Grazul-Bilska AT, Johnson ML, Reynolds LP, Redmer DA (2006) Isolation and characterization of ovine luteal pericytes and effects of nitric oxide on pericyte expression of angiogenic factors. Endocrine 29: 467–476. 16943586 10.1385/endo:29:3:467

[6] Darland DC, Massingham LJ, Smith SR, Piek E, Saint-Geniez M, D'Amore PA (2003) Pericyte production of cell-associated VEGF is differentiation-dependent and is associated with endothelial survival. Dev Biol 264: 275–288. 14623248 10.1016/j.ydbio.2003.08.015

[7] Kashiwamura Y, Sano Y, Abe M, Shimizu F, Haruki H, Maeda T, et al (2011) Hydrocortisone enhances the function of the blood-nerve barrier through the up-regulation of claudin-5. Neurochem Res 36: 849–855. doi: 10.1007/s11064-011-0413-6 21293925 10.1007/s11064-011-0413-6

[8] Watanabe S, Morisaki N, Tezuka M, Fukuda K, Ueda S, Koyama N, et al (1997) Cultured retinal pericytes stimulate in vitro angiogenesis of endothelial cells through secretion of a fibroblast growth factor-like molecule. Atherosclerosis 130: 101–107. 9126653 10.1016/s0021-9150(96)06050-9

[9] Svensson A, Ozen I, Genove G, Paul G, Bengzon J (2015) Endogenous brain pericytes are widely activated and contribute to mouse glioma microvasculature. PLoS One 10: e0123553 doi: 10.1371/journal.pone.0123553 25875288 10.1371/journal.pone.0123553 PMC4395339

[10] Ozen I, Deierborg T, Miharada K, Padel T, Englund E, Genove G, et al (2014) Brain pericytes acquire a microglial phenotype after stroke. Acta Neuropathol 128: 381–396. doi: 10.1007/s00401-014-1295-x 24848101 10.1007/s00401-014-1295-x PMC4131168

[11] Gonul E, Duz B, Kahraman S, Kayali H, Kubar A, Timurkaynak E (2002) Early pericyte response to brain hypoxia in cats: an ultrastructural study. Microvasc Res 64: 116–119. doi: 10.1006/mvre.2002.2413 12074637 10.1006/mvre.2002.2413

[12] Gidday JM (2006) Cerebral preconditioning and ischaemic tolerance. Nat Rev Neurosci 7: 437–448. doi: 10.1038/nrn1927 16715053 10.1038/nrn1927

[13] Robin ED, Murphy BJ, Theodore J (1984) Coordinate regulation of glycolysis by hypoxia in mammalian cells. J Cell Physiol 118: 287–290. doi: 10.1002/jcp.1041180311 6699103 10.1002/jcp.1041180311

[14] Semenza GL (2001) HIF-1, O(2), and the 3 PHDs: how animal cells signal hypoxia to the nucleus. Cell 107: 1–3. 11595178 10.1016/s0092-8674(01)00518-9

[15] Pugh CW, Ratcliffe PJ (2003) Regulation of angiogenesis by hypoxia: role of the HIF system. Nat Med 9: 677–684. doi: 10.1038/nm0603-677 12778166 10.1038/nm0603-677

[16] Levy DE, Darnell JE (2002) STATs: transcriptional control and biological impact. Nat Rev Mol Cell Biol 3: 651–662. doi: 10.1038/nrm909 12209125 10.1038/nrm909

[17] Jung JE, Kim GS, Chan PH (2011) Neuroprotection by interleukin-6 is mediated by signal transducer and activator of transcription 3 and antioxidative signaling in ischemic stroke. Stroke 42: 3574–3579. doi: 10.1161/STROKEAHA.111.626648 21940958 10.1161/STROKEAHA.111.626648 PMC3395465

[18] Gertz K, Kronenberg G, Kalin RE, Baldinger T, Werner C, Balkaya M, et al (2012) Essential role of interleukin-6 in post-stroke angiogenesis. Brain 135: 1964–1980. doi: 10.1093/brain/aws075 22492561 10.1093/brain/aws075 PMC3359750

[19] Sun Y, Jin K, Xie L, Childs J, Mao XO, Logvinova A, et al (2003) VEGF-induced neuroprotection, neurogenesis, and angiogenesis after focal cerebral ischemia. J Clin Invest 111: 1843–1851. doi: 10.1172/JCI17977 12813020 10.1172/JCI17977 PMC161428

[20] Jung JE, Lee HG, Cho IH, Chung DH, Yoon SH, Yang YM, et al (2005) STAT3 is a potential modulator of HIF-1-mediated VEGF expression in human renal carcinoma cells. FASEB J 19: 1296–1298. doi: 10.1096/fj.04-3099fje 15919761 10.1096/fj.04-3099fje

[21] Gray MJ, Zhang J, Ellis LM, Semenza GL, Evans DB, Watowich SS, et al (2005) HIF-1alpha, STAT3, CBP/p300 and Ref-1/APE are components of a transcriptional complex that regulates Src-dependent hypoxia-induced expression of VEGF in pancreatic and prostate carcinomas. Oncogene 24: 3110–3120. doi: 10.1038/sj.onc.1208513 15735682 10.1038/sj.onc.1208513

[22] Jung JE, Kim HS, Lee CS, Shin YJ, Kim YN, Kang GH, et al (2008) STAT3 inhibits the degradation of HIF-1alpha by pVHL-mediated ubiquitination. Exp Mol Med 40: 479–485. doi: 10.3858/emm.2008.40.5.479 18985005 10.3858/emm.2008.40.5.479 PMC2679355

[23] Paul G, Özen I, Christophersen NS, Reinbothe T, Bengzon J, Visse E, et al (2012) The Adult Human Brain Harbors Multipotent Perivascular Mesenchymal Stem Cells. PLOS ONE 7: e35577 doi: 10.1371/journal.pone.0035577 22523602 10.1371/journal.pone.0035577 PMC3327668

[24] Paul G, Özen I, Christophersen N, Bengzon J, Visse E, Jansson K, et al (2012) The adult human brain harbors multipotent perivascular mesenchymal stem cells PLoS ONE 7. 10.1371/journal.pone.0035577 PMC3327668 22523602

[25] Gaceb A, Ozen I, Padel T, Barbariga M, Paul G (2017) Pericytes secrete pro-regenerative molecules in response to platelet-derived growth factor-BB. J Cereb Blood Flow Metab: 271678X17719645. 10.1177/0271678X17719645 PMC5757443 28741407

[26] Ruscher K, Shamloo M, Rickhag M, Ladunga I, Soriano L, Gisselsson L, et al (2011) The sigma-1 receptor enhances brain plasticity and functional recovery after experimental stroke. Brain 134: 732–746. doi: 10.1093/brain/awq367 21278085 10.1093/brain/awq367

[27] Irizarry RA, Hobbs B, Collin F, Beazer-Barclay YD, Antonellis KJ, Scherf U, et al (2003) Exploration, normalization, and summaries of high density oligonucleotide array probe level data. Biostatistics 4: 249–264. doi: 10.1093/biostatistics/4.2.249 12925520 10.1093/biostatistics/4.2.249

[28] Stacklies W, Redestig H, Scholz M, Walther D, Selbig J (2007) pcaMethods—a bioconductor package providing PCA methods for incomplete data. Bioinformatics 23: 1164–1167. doi: 10.1093/bioinformatics/btm069 17344241 10.1093/bioinformatics/btm069

[29] Chen H, Boutros PC (2011) VennDiagram: a package for the generation of highly-customizable Venn and Euler diagrams in R. BMC Bioinformatics 12: 35 doi: 10.1186/1471-2105-12-35 21269502 10.1186/1471-2105-12-35 PMC3041657

[30] Mootha VK, Lindgren CM, Eriksson KF, Subramanian A, Sihag S, Lehar J, et al (2003) PGC-1alpha-responsive genes involved in oxidative phosphorylation are coordinately downregulated in human diabetes. Nat Genet 34: 267–273. doi: 10.1038/ng1180 12808457 10.1038/ng1180

[31] Subramanian A, Tamayo P, Mootha VK, Mukherjee S, Ebert BL, Gillette MA, et al (2005) Gene set enrichment analysis: a knowledge-based approach for interpreting genome-wide expression profiles. Proc Natl Acad Sci U S A 102: 15545–15550. doi: 10.1073/pnas.0506580102 16199517 10.1073/pnas.0506580102 PMC1239896

[32] Consortium EP (2012) An integrated encyclopedia of DNA elements in the human genome. Nature 489: 57–74. doi: 10.1038/nature11247 22955616 10.1038/nature11247 PMC3439153

[33] Sloan CA, Chan ET, Davidson JM, Malladi VS, Strattan JS, Hitz BC, et al (2016) ENCODE data at the ENCODE portal. Nucleic Acids Res 44: D726–732. doi: 10.1093/nar/gkv1160 26527727 10.1093/nar/gkv1160 PMC4702836

[34] Schodel J, Oikonomopoulos S, Ragoussis J, Pugh CW, Ratcliffe PJ, Mole DR (2011) High-resolution genome-wide mapping of HIF-binding sites by ChIP-seq. Blood 117: e207–217. doi: 10.1182/blood-2010-10-314427 21447827 10.1182/blood-2010-10-314427 PMC3374576

[35] Afgan E, Baker D, van den Beek M, Blankenberg D, Bouvier D, Cech M, et al (2016) The Galaxy platform for accessible, reproducible and collaborative biomedical analyses: 2016 update. Nucleic Acids Res 44: W3–W10. doi: 10.1093/nar/gkw343 27137889 10.1093/nar/gkw343 PMC4987906

[36] Liu T, Ortiz JA, Taing L, Meyer CA, Lee B, Zhang Y, et al (2011) Cistrome: an integrative platform for transcriptional regulation studies. Genome Biol 12: R83 doi: 10.1186/gb-2011-12-8-r83 21859476 10.1186/gb-2011-12-8-r83 PMC3245621

[37] Thomas PD, Campbell MJ, Kejariwal A, Mi H, Karlak B, Daverman R, et al (2003) PANTHER: A Library of Protein Families and Subfamilies Indexed by Function. Genome Research 13: 2129–2141. doi: 10.1101/gr.772403 12952881 10.1101/gr.772403 PMC403709

[38] Livak KJ, Schmittgen TD (2001) Analysis of Relative Gene Expression Data Using Real-Time Quantitative PCR and the 2−ΔΔCT Method. Methods 25: 402–408. doi: 10.1006/meth.2001.1262 11846609 10.1006/meth.2001.1262

[39] Brugè F, Venditti E, Tiano L, Littarru GP, Damiani E (2011) Reference gene validation for qPCR on normoxia- and hypoxia-cultured human dermal fibroblasts exposed to UVA: Is β-actin a reliable normalizer for photoaging studies? Journal of Biotechnology 156: 153–162. doi: 10.1016/j.jbiotec.2011.09.018 21963587 10.1016/j.jbiotec.2011.09.018

[40] Schreiber E, Matthias P, Muller MM, Schaffner W (1989) Rapid detection of octamer binding proteins with 'mini-extracts', prepared from a small number of cells. Nucleic Acids Res 17: 6419 2771659 10.1093/nar/17.15.6419 PMC318318

[41] Hoffmann CJ, Harms U, Rex A, Szulzewsky F, Wolf SA, Grittner U, et al (2015) Vascular signal transducer and activator of transcription-3 promotes angiogenesis and neuroplasticity long-term after stroke. Circulation 131: 1772–1782. doi: 10.1161/CIRCULATIONAHA.114.013003 25794850 10.1161/CIRCULATIONAHA.114.013003

[42] Yu P, Wilhelm K, Dubrac A, Tung JK, Alves TC, Fang JS, et al (2017) FGF-dependent metabolic control of vascular development. Nature 545: 224–228. doi: 10.1038/nature22322 28467822 10.1038/nature22322 PMC5427179

[43] Wang S, Li M, Zhang W, Hua H, Wang N, Zhao J, et al (2017) Growth differentiation factor 15 promotes blood vessel growth by stimulating cell cycle progression in repair of critical-sized calvarial defect. Sci Rep 7: 9027 doi: 10.1038/s41598-017-09210-4 28831101 10.1038/s41598-017-09210-4 PMC5567281

[44] Wen Z, Zhong Z, Darnell JE Jr. Maximal activation of transcription by statl and stat3 requires both tyrosine and serine phosphorylation. Cell 82: 241–250. 7543024 10.1016/0092-8674(95)90311-9

[45] Frost J, Galdeano C, Soares P, Gadd MS, Grzes KM, Ellis L, et al (2016) Potent and selective chemical probe of hypoxic signalling downstream of HIF-alpha hydroxylation via VHL inhibition. Nat Commun 7: 13312 doi: 10.1038/ncomms13312 27811928 10.1038/ncomms13312 PMC5097156

[46] Yoon S, Woo SU, Kang JH, Kim K, Shin HJ, Gwak HS, et al (2012) NF-[kappa]B and STAT3 cooperatively induce IL6 in starved cancer cells. Oncogene 31: 3467–3481. doi: 10.1038/onc.2011.517 22105366 10.1038/onc.2011.517

[47] Schwaninger M, Neher M, Viegas E, Schneider A, Spranger M (1997) Stimulation of interleukin-6 secretion and gene transcription in primary astrocytes by adenosine. J Neurochem 69: 1145–1150. 9282937 10.1046/j.1471-4159.1997.69031145.x

[48] Gough DJ, Koetz L, Levy DE (2013) The MEK-ERK Pathway Is Necessary for Serine Phosphorylation of Mitochondrial STAT3 and Ras-Mediated Transformation. PLOS ONE 8: e83395 doi: 10.1371/journal.pone.0083395 24312439 10.1371/journal.pone.0083395 PMC3843736

