# Supplementary figures and images for: STAT3 precedes HIF1α transcriptional responses to oxygen and oxygen and glucose deprivation in human brain pericytes
Source: PLoS One. 2018 Mar 8;13(3):e0194146. doi: 10.1371/journal.pone.0194146 (PMC5843348; doi:10.1371/journal.pone.0194146)

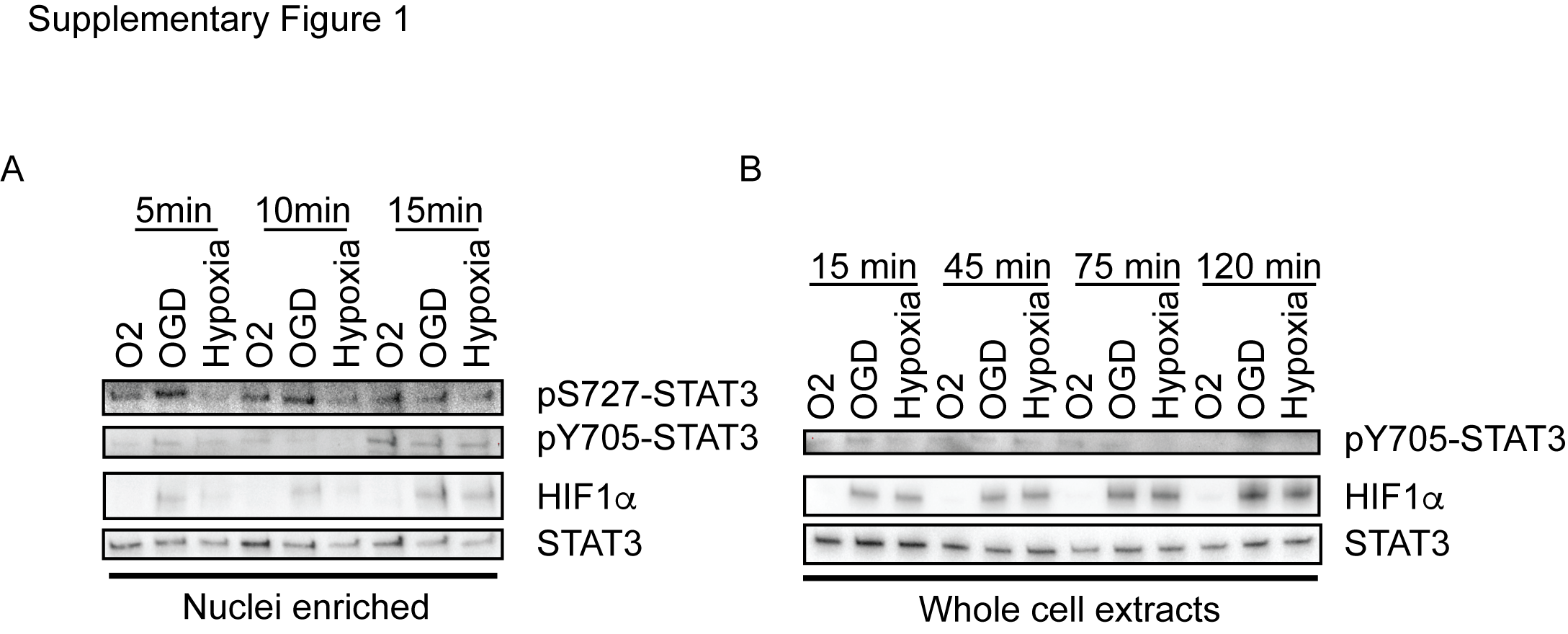

Supplement: S1 Fig — Western blot of (A) nuclear extracts (B) or whole cell extracts from Sciencell pericytes treated with normoxia, hypoxia or OGD. (A) 5,10 or 15 min treatments followed by extraction of nuclear proteins. In (A) anti-pS727STAT3 was used to visualise nuclear phosphorylation of STAT3 required for transcriptional activity. PY705 was used to measure the nuclear phosphorylation required for dimerisation and nuclear translocation of STAT3. Nuclear anti-HIF1α was measured at 5, 10 or 15 minutes of treatments. Anti-STAT3 indicated the steady state nonphosphorylated STAT3 in the nuclear extracts and was compared to the pS727- and pY705-STAT3 levels. (B) Pericytes treated with normoxia, hypoxia or OGD for 15, 45, 75 or 120min prior to whole cell lysis. Western blot of pY705-STAT3, HIF1α or total STAT3. (TIF) [file pone.0194146.s001.tif]
